# A Case of Ruptured Pulmonary Hydatid Cyst of the Liver and Review of the Literature

**DOI:** 10.1155/2017/7639056

**Published:** 2017-09-24

**Authors:** Nilufer Bulut, Sevinc Dagıstanlı

**Affiliations:** ^1^Department of Medical Oncology, Kanuni Sultan Suleyman Education and Research Hospital, Istanbul, Turkey; ^2^Department of Surgery, Division of General Surgery, Kanuni Sultan Suleyman Education and Research Hospital, Istanbul, Turkey

## Abstract

**Background:**

Hydatid cyst is an endemic disease frequently localized to the liver. It is frequently observed in Southeast Europe, Middle East, and Turkey. Although the cyst rupture can occur spontaneously, it can also occur upon albendazole treatment. Its surgical treatment includes cystotomy, capitonnage, and wedge resection.

**Material-Method:**

A 56-year-old male immigrant was admitted with fever, pain, and cough. Albendazole treatment was initiated and elective surgery was planned. Upon his admission to emergency service, he was diagnosed with pneumonia, and a spontaneous cyst rupture was detected.

**Result:**

Thoracotomy and cystotomy were performed. Bile leakage aspiration and lung wedge resection were also performed.

**Conclusion:**

Different surgical methods are used in the treatment of hydatid cysts depending on the localization and complications. Follow-up with antihelminthic drugs such as albendazole and mebendazole is recommended in medical treatment.

## 1. Introduction

Hydatid cyst is a parasitic disease caused by* Echinococcus granulosus* which is most frequently seen in the liver, followed by the lungs [1, 2]. It is endemic in Turkey with a prevalence of 50 to 400 in every 100.000 individuals and an incidence of 5.7 in every 100.000 individuals [3, 4].

Cyst hydatid may remain symptomless for years but also may show pressure symptoms due to the growth, rupture to the neighboring structures, or becoming contaminated with infection. In our case, the hepatic cyst hydatid, which did not cause anaphylaxis despite being ruptured, was treated.

## 2. Case Report

**Figure 1:**
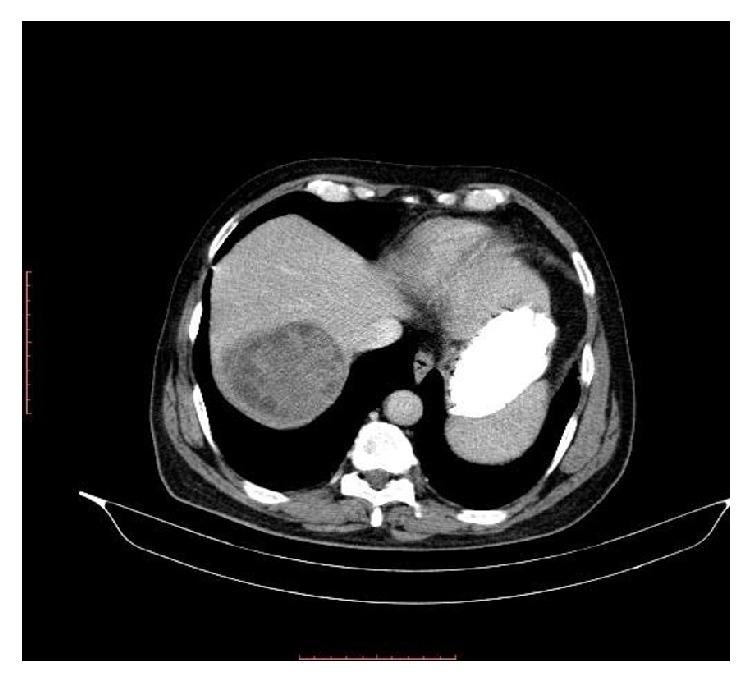
Hydatid cyst in right lobe liver on CT scan.

**Figure 2:**
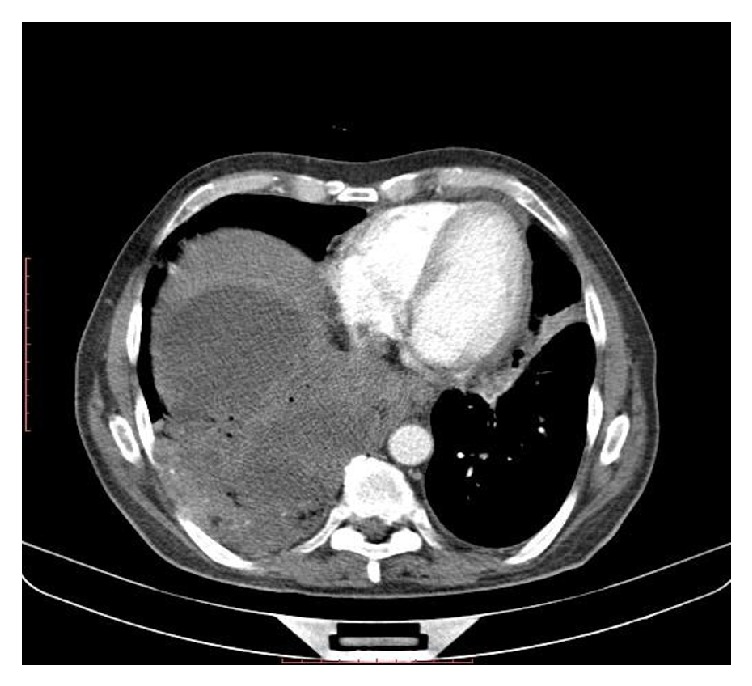
Ruptured hydatid cyst in lung via diaphragm on CT scan.

**Figure 3:**
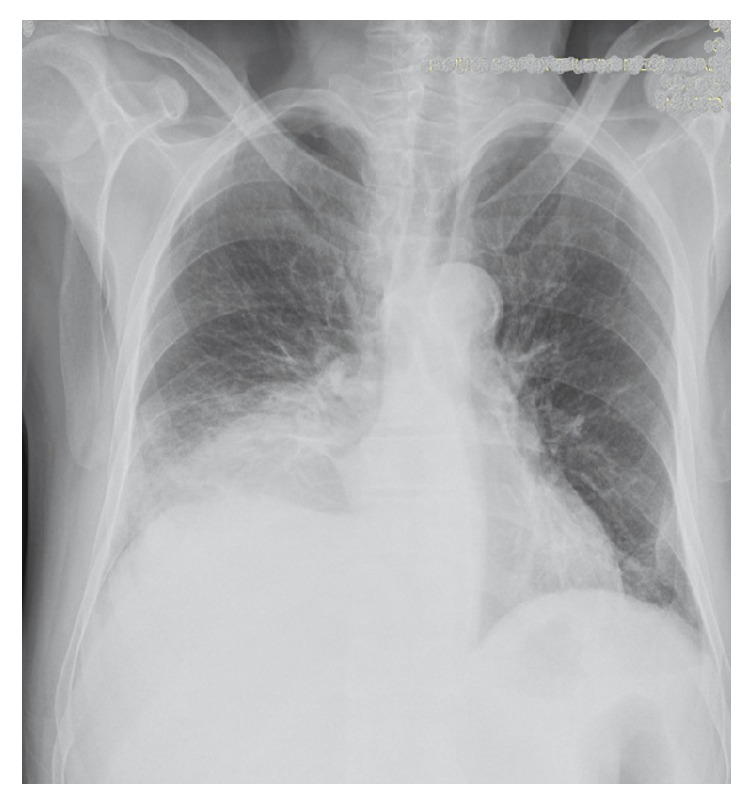
Pneumonic infiltration on chest X-ray.

**Figure 4:**
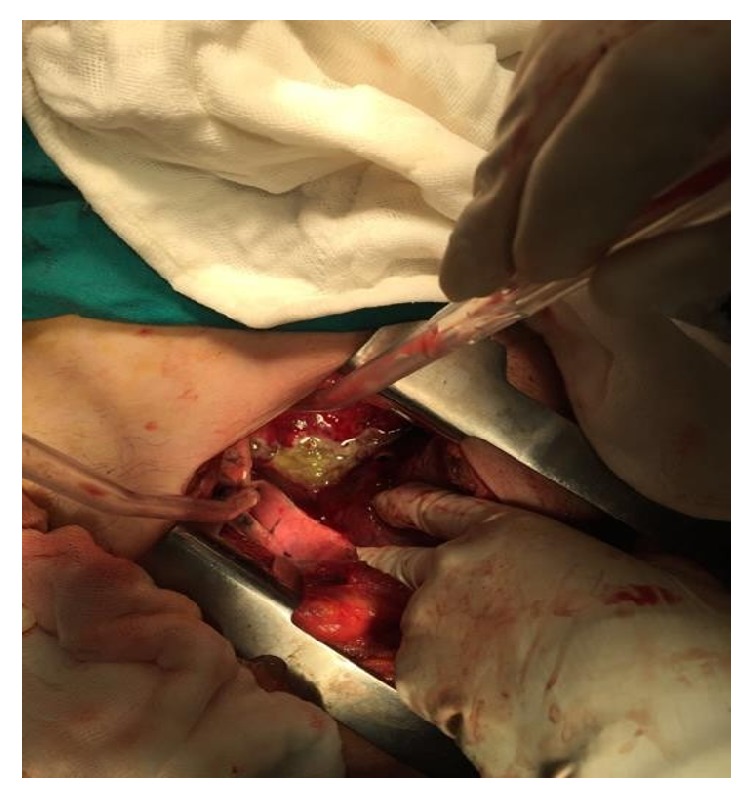
Lung cystotomy with subcostal incision.

**Figure 5:**
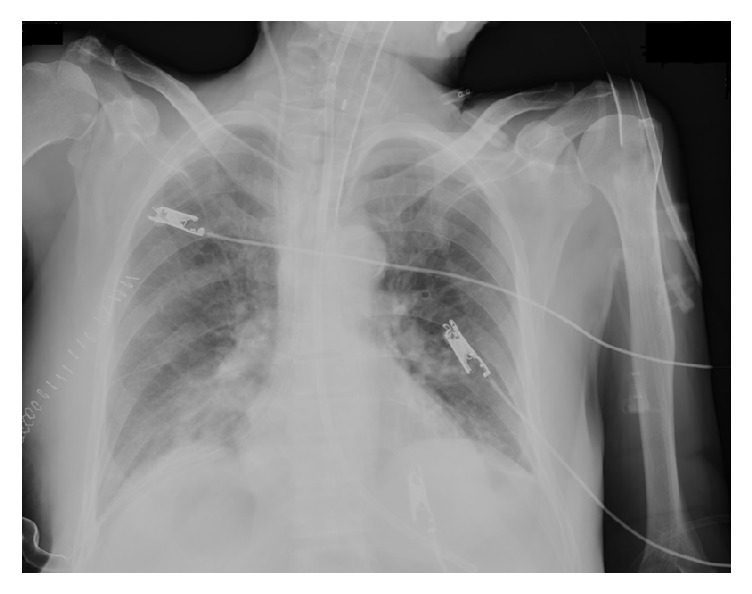
Complete expansion of the lung after the thorax tube was removed.

A 56-year-old Syrian male immigrant was admitted with fever, shortness of breath, and abdominal pain in the upper right quadrant for two months. His medical history revealed no known comorbidities. Emergency surgical examination did not reveal any rebound, muscular defence in the abdomen. Laboratory test results were as follows: white blood cell count (WBC) 13.000/mm^3^ and C-reactive protein (CRP) 232 mg/dL tomography showed echinococcosis cyst (CE) 102 × 92 mm at the liver segments 6-7. Abdominal ultrasonography revealed a CE-4 type cystic lesion of 106 × 70 mm at the right lobe (Figures 1 and 2). The patient was treated with albendazole (800 mg/day), considering the risk of abscess due to long-term abdominal pain and fever, with metronidazole. However, the patient who admitted to the emergency service the next day with shortness of breath was hospitalized with a diagnosis of pneumonia. In the posteroanterior X-ray examination, a consolidation was observed in the right basal segment of the lung (Figure 3). He was treated with ceftriaxone 2 g/day and clarithromycin 500 mg/day. Oxygen saturation was 97%, arterial blood pressure was 105/65 mmHg, and pulse was 80/min. No rale or rhoncus was determined during the auscultation. We concluded that the rupture of the hydatid cyst was responsible for the clinical condition of the patient and planned drainage of the lung infection with an abdominal incision. As the clinical condition of the patient deteriorated and WBC count gradually increased to 22,700/mm3 and CRP to 314 mg/dl, abdominal cavity was entered with a subcostal incision. A grade 3 hydatid cyst with a solid content and secondary cysts was determined. It was extending to the thoracic cavity through the detachment of the laminar membrane. There was no rupture to the bronchial structures. As the cyst was attached to the diaphragm with a large surface, cystectomy could not be carried out in the liver. Due to the multiple millimetric perforations in the diaphragm, thoracotomy was performed. The cyst was ruptured to the thoracic cavity via diaphragmatic invasion (Figure 4). Cystotomy was also performed to remove the cyst localized in the liver and lung, and millimetric perforations in the diaphragm were primarily repaired. A drainage tube was placed in the abdomen and a thoracic tube was placed in the pleural cavity. The patient was followed in the intensive care unit. During follow-up, his blood chemistry values were as follows: WBC: 28.000/mm^3^, CRP: 27 mg/dL, and procalcitonin: 3.38 ng/mL. Twelve days after the operation, biliary content was observed in endotracheal aspiration, and bronchopleural fistula was suspected. Therefore, rethoracotomy and lung wedge resection were performed. One week later, the thoracic tube was removed due to complete expansion of the lung and absence of air leakage (Figure 5). Repeated abdominal ultrasound showed that 120 mL of biliary fluid was aspirated by the injection from the debrided cyst in the liver segment 7. The WBC regressed to 7400/mm^3^ and CRP to 15 mg/dL. The patient was discharged on 15th day. In the histopathological examination, necrotic cuticular membrane pieces and bile in the cyst lumen were detected.

## 3. Discussion

Hydatid cyst rupture can be spontaneous, iatrogenic, or traumatic. Rupture of hydatid cyst in diaphragmatic location is rare. Coughing, dyspnea, fever, hemoptysis, flank pain, chest pain, and secondary pneumonia with pressure to the neighboring bronchi can develop due to the pulmonary rupture of the hydatid cyst on the diaphragmatic surface of the liver [5].

Bronchopneumonia, similar to our case, can develop due to hydatid cyst of the lung, or it can be asymptomatic. While secondary infections develop due to the rupture of the cyst (in 5–8% of the cases), if the cyst fluid enters to the blood circulation of the host, anaphylaxis and allergic reactions can occur [6–8].

Primary lung hydatid cysts can be asymptomatic, as well as spontaneously ruptured or infected. Treatment options include surgical draining of the cyst, peeling of the germinative membrane, and, for inoperable patients, albendazole treatment [1]. Spontaneous or albendazole treatment-related ruptures can be seen in many cases. In some cases, anaphylaxis may not be present due to spontaneous narrowing, although the cyst is ruptured [1, 5]. Moreover, recovery is observed in patients receiving albendazole treatment instead of undergoing surgery, despite the ruptured cyst [1, 4]. For hydatid cyst of the lung, the location of the cyst is critical for the choice of treatment. For centrally localized cysts, as the cyst can lead to bronchopulmonary tree, surgical drainage of the cyst and capitonnage is performed, whereas in asymptomatic cysts surrounded by parenchyma, lobectomy, wedge resection, pericystectomy, endocystectomy, or combined albendazole treatment is preferred. Using these for at least 3 months can reduce the number of scolices [1, 5]. However, there is no consensus on the duration of medical treatment. The treatment can last for at least one month to at most one year [7].

In our case, capitonnage was not performed, since the cyst was close to diaphragmatic surface, and bile leakage was observed during follow-up. Later, wedge resection was performed in the lung, due to the localization of the cyst. Hydatid cyst in the liver was attached to the diaphragmatic surface and cystotomy was performed. Bile accumulation in the liver pouch occurred, as omentopexy was not performed. Bilious content was aspirated and the area was washed with saline. Albendazole treatment (800 mgr/day) was continued for three months.

Spontaneous ruptures can occur due to trauma or increase in the pressure of the growing cyst [2]. Upon its rupture, according to the associated organ, the cyst causes anaphylactic reaction such as pain and fever, hypotension, and fatigue [7, 8]. Although these findings primarily suggest pneumonia, sudden changes in the overall state, persistent cough, and hypotension with the respiratory distress suggest anaphylaxis [3]. In particular, chest X-ray shows cavitary lesions containing air and lotus flower or meniscus appearance. It forms hyperdense areas with an icy glass appearance through direct invasion depending on the defects on the diaphragmatic side of the liver. Diagnosis of the ruptured cyst is made during surgery, via detection of the cavity and identification of the defect on the degenerate laminar membrane and diaphragmatic surface. In such cases, thoracotomy, laparotomy, or mixed surgical interventions; that is, thoracophrenolaparotomy techniques can be performed. The primary or prosthetic repair of the diaphragm is completed with surgical interventions like cystectomy, cystotomy, and capitonnage or omentoplasty [9].

## 4. Conclusion

In conclusion, a ruptured hydatid cyst may present asymptomatically or with an anaphylactic reaction. In symptomatic cases, depending on the organ where the cyst is located, surgical excision and capitonnage can be performed. Primary lesion at the dome of the liver can be ruptured into the lung through the diaphragmatic route. In such cases, wedge resection and cystotomy to the primary region are performed, and recurrences are followed with albendazole treatment.
